# Human factors and systems simulation methods to optimize peri-operative EHR design and implementation

**DOI:** 10.1186/s41077-025-00349-z

**Published:** 2025-04-23

**Authors:** Mirette Dubé, Jonathan D. Hron, Susan Biesbroek, Myrna Chan-MacRae, AEliot Shearer, Rocco Landi, Melanie Swenson, Daniel J. Kats, Doreen White, Reilly Birmingham, Lauren Coogle, Jennifer Arnold

**Affiliations:** 1 Healthcare Systems Simulation International Inc, 51 GlenEagles Terrace, Cochrane, AB T4C 1W4 Canada; 2 Human Factors and Systems Design, Boston Children’s Hospital, 300 Longwood Avenue, Boston, MA 02115 USA; 3 Boston Children’s Hospital, 300 Longwood Avenue, Boston, MA 02115 USA; 4 Harvard Medical School, 330 Brookline Avenue, Boston, MA 02215 USA; 5 Department of Otolaryngology and Communication Enhancement, Boston Children’s Hospital, 300 Longwood Avenue, Boston, MA 02115 USA; 6 Department of Otolaryngology Head and Neck Surgery, Harvard Medical School, 330 Brookline Avenue, Boston, MA 02215 USA; 7 Department of Anesthesiology, Critical Care and Pain Medicine, Medical Director of Health Technology Management, Boston Children’s Hospital, 300 Longwood Avenue, Boston, MA 02115 USA; 8 Boston Children’s Hospital, 9 Hope Ave, Waltham, MA 02453 USA; 9 Children’s Hospital of Philadelphia, 3401 Civic Center Blvd, Philadelphia, PA 19104 USA; 10 Immersive Design Systems, Boston Children’s Hospital, 300 Longwood Avenue, Boston, MA 02115 USA

**Keywords:** Systems simulation, Simulation, Human factors, Electronic health record, Information technology, Healthcare, Usability testing, Patient safety, Quality improvement

## Abstract

The increase in adoption of Electronic Health records (EHR) in healthcare can be overwhelming to users and pose hidden safety threats and inefficiencies if the system is not well aligned with workflows. This quality improvement study, facilitated from September 2023–April 2024, aimed to proactively test a new EHR using systems focused simulation and Human factors methods, prior to go-live, in a peri-operative children’s hospital setting to improve safety, efficiency and usability of the EHR. The project was conducted at a large, academic, quaternary care children’s hospital undergoing a transition from one EHR to another. Two cycles of usability testing followed by in situ simulations focused on testing the new EHR with interprofessional peri-operative team members prior to go live. Usability testing, using relevant clinical workflows, was completed over zoom using the EHR “testing” environment with individual care providers across multiple peri-operative roles. In situ simulations were facilitated in the actual peri-operative and Otolaryngology clinic spaces with full interprofessional teams. Qualitative data was collected and summarized through debriefing and recordings of the sessions. Human factors and patient safety principles were integrated throughout the recommendations. A total of 475 recommendations were made to improve the safety, efficiency, usability, and optimization of the EHR. The outcomes included a range of usability and system issues including latent safety threats and their impact on safe and quality patient care. There was a plethora of usability improvements, including some critical issues that were uncovered and mitigated prior to the go live date.

## Background

In the past 15 years, there has been an increasing adoption of Electronic Health Records (EHR) [1–3]. These electronic systems are implemented across hospitals or health systems, requiring attention to staff training, project and change management, and especially patient and staff safety [4–8]. In complex health care systems, adding a significant change (i.e., new EHR) can be overwhelming for users, pose safety risks to patients, and may cause inefficiencies if the systems’ design does not align with current workflows [9–13]. These factors can lead to low adoption rates and increased dissatisfaction among users. EHRs have helped healthcare institutions reduce medical costs, track and share patient information easier across hospital sites, and reduce medication errors while eliminating illegible handwritten documentation [14–19]. In the longer term, they may reduce the extra steps required to navigate multiple documentation systems, when used.

Human factors (HF) and systems focused simulation (SFS) applications involve the scientific study and applications of how people interact with the equipment, tools and technology, information, and other people to perform tasks in their environment [20–23]. As described in the Systems Engineering Initiative for Patient Safety (SEIPS 2.0) human factors framework, any change to system elements, such as technology (i.e., a new EHR), tools, environment, tasks, and so on, can have unpredictable impact to other system elements, healthcare processes, and desired outcomes including safe patient care [24–26]. Healthcare simulation has been used as an effective tool to uncover latent safety threats in healthcare environments, systems of care, and technologies as it can bridge the delta between work as imagined and work as done [27, 28]. While a new EHR is built with safety and efficiency in an ideal state of work as imagined, what really happens in application during actual patient care can be very different resulting in unintended consequences [29]. Simulation ensures an experiential approach focused on user centred design where users participate in methods such as usability testing or in situ simulations (i.e., simulation located in the actual clinical environment) and then provide feedback through debriefing [30–33]. The debriefing is focused on identifying and mitigating hazards related to the new or changing system element, not on the performance, knowledge, or skills of clinicians.

HF methodologies, including usability testing, are essential to integrate during the design, development, implementation, and operation of any eHealth system to protect patients against harm [34–36]. Usability issues can account for up to 60% of information technology (IT) sentinel events according to the Joint Commission [37, 38], and the introduction of EHRs can lead to unintended consequences [14, 34, 35, 39]. Given that approximately 3–5 end users may identify up to 85% of the usability issues in a product, usability testing is a cost-effective method for identification of potential risks and challenges in human-product interaction [20].

New EHR systems are often generic or “out of the box” and then customized to meet the needs of specific institutions but are typically not tested prior to implementation. Customization typically involves demonstrations and working group sessions led by analysts who are competent in the system. Without detailed “hand to keyboard” scenarios that mimic real life work, it can be difficult to identify EHR build issues, including deviations from current workflows and potential errors [40].

Enhancing the build process with cycles of usability testing and in situ systems-focused simulations enables many benefits, including improving the design, safety, and efficiency of the system while engaging end users and enhancing adoption [41]. The peri-operative environment is especially susceptible to high-risk safety threats, staff anxiety, and decreased efficiency given the environment, frequent EHR interaction, and fast paced workflows.

Our paper demonstrates a quality improvement (QI) study utilizing HF and SFS methods to improve the usability of a new EHR for perioperative care providers.

## Methods

### Setting

The project was conducted at a large, academic, quaternary care children’s hospital undergoing a transition from one EHR to another.

This project was deemed Quality improvement (QI) by the *Boston Children’s Hospital* Department of Pediatrics Performance Excellence Group. QI projects that are designed to improve clinical care to better conform to established or accepted standards are considered exempt from human subject’s review by our institutional review board.

### Project team

The project team consisted of members from the hospital’s IT and simulation programs’ leadership, EHR implementation team (including analysts and frontline clinicians), and an external consultancy group that provided expertise in HF and SFS.

### Needs assessment

Peri-operative care was identified as a focus for HF and SFS due to the time-sensitivity during patient care, complexity of workflows, and high volume of throughput suggesting proactive evaluation for safety and efficiency as highly beneficial. During project planning, the study team engaged surgeons, physician assistants, perioperative nurses, business operations, and scheduling roles in a video conference change-based needs assessment [22]. Based on case types and availability, the Otolaryngology (ORL) department was chosen to represent the surgical perspective. Based on participants’ greatest areas of concern, level of change from current workflows, and potential gaps they anticipated with the new EHR, the scenario and testing content was identified: tympanostomy tube placement surgery, with and without tonsillectomy/adenoidectomy.

### Plan, do, study, act cycles

**Fig. 1:**
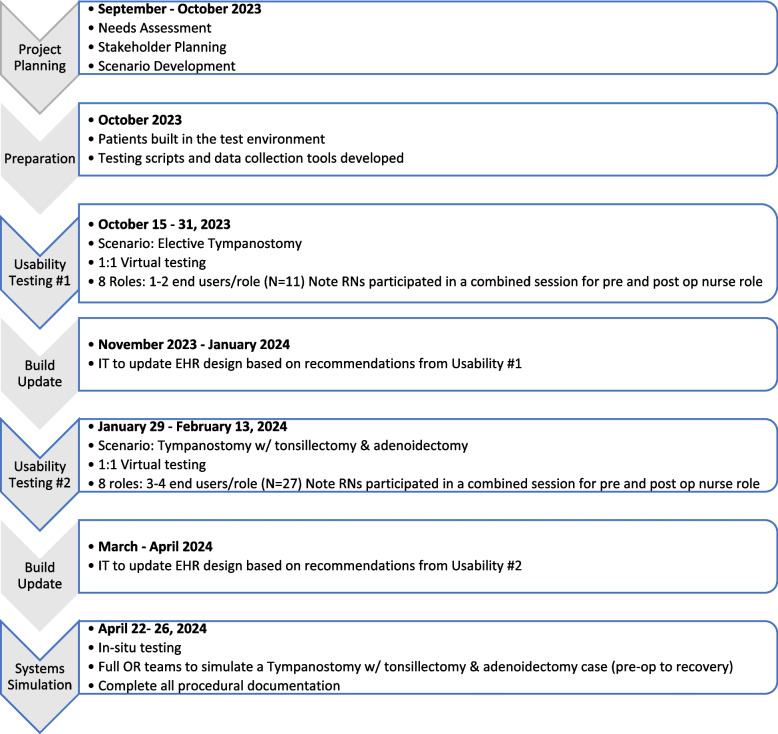
Project timeline. Providing an overview of the project timeline and key activities

Two cycles of 1:1 participant usability testing (Plan, Do, Study, Act cycles) of the EHR using typical workflows for each role were used during usability testing, and later team based in-situ simulation phases**.** Figure 1 depicts a detailed timeline of each cycle of usability testing and simulations.

### Usability testing

The initial plan was to complete a larger test cycle of 3–4 participants/role in October of 2023 (cycle 1) and a smaller retest cycle in January/February of 2024 (cycle 2) to validate build changes. Due to delays in the build, this plan was reversed so that cycle 1 of usability testing included 1–2 participants/role. As the EHR build progressed and approached the implementation date, cycle 2 (January/February 2024) increased the number participants to enable a larger cohort of 3–4 participants/role to participate in the usability testing (Fig. 1).

All usability testing sessions were facilitated by SFS and HF experts, lasting for 90–120 min via video conference and were recorded for data analysis purposes with consent. Each session included a prebrief with simulated scenarios in the testing EHR environment.

Each prebrief followed simulation best practice, including welcome/introductions, rationale for usability and simulation testing, goals of the session (i.e., testing the system to collect user feedback and not the person’s abilities or knowledge of the system), provided updates from improvement work completed, and key messages to build psychological safety, transparency, commitment to respect time, and anonymize participant feedback [42]. The workflow and scenario was introduced with a short orientation to the EHR interface.

Each participant completed their specific documentation workflows for the given scenario (Table 1).

**Table 1:** Roles, usability testing cycles 1 and 2: workflow steps and objectives. Cycle 1 scenario: tympanostomy. Cycle 2 scenario: tympanostomy with tonsillectomy and adenoidectomy

Role (# participants/cycle)	Workflow steps	Objectives
**ORL Surgeon** (Cycle 1 *n* = 1) (Cycle 2 *n* = 3) **Physician Assistant** (Cycle 1 *n* = 1) (Cycle 2 *n* = 4)	• Patient arrives for a surgical consult visit • Progress note (HPI, Physical Exam) • Place case request for **surgery (Cycle 2 = two procedures)** • Place pre-op and post-op orders for the appropriate phase of care • Obtain surgical consent	• Progress note functionality and design • Consent form design and use • Pre-op order sets/panels • Preference card build
**Otolaryngology Scheduler** (Cycle 1 *n* = 2) (Cycle 2 *n* = 4)	• Review the scheduling depot information • Review the case request • Approve the case request/mark it ‘ready to schedule’	• Ensure completeness of the request • Communication with 2nd scheduler (OR scheduler)
**Operating Room Scheduler** (Cycle 1 *n* = 2) (Cycle 2 *n* = 4)	• Review the scheduling depot information • Review the case request • Update case panel lengths • Book the surgical case from the scheduling tool	• Preference card/panel length accuracy • Communication with Otolaryngology scheduler • Ease of scheduling and information available in the depot views
**Pre-op Nurse** (Cycle 1 *n* = 2 (Cycle 2 *n* = 4) *participants completed both pre-op and post-op nursing tasks	• Moves patient into the Pre-op department within the system and assigns them a bed • Release orders • Complete pre-op documentation (i.e., vitals, exam, inventory of patient belongings) • Review consent form • Complete the pre-op checklist	• Patient movement • Content and flow of the assessment forms • Content and build of the checklist
**Anesthesiologist** (Cycle 1 *n* = 1) (Cycle 2 *n* = 4)	• Review the history • Review the pre-op documentation and pre-evaluation • Place post-op orders for recovery • Create the Anesthesia note • Obtain consent • Move patient into intra-procedure • Anesthesia Checklist • Intra-procedure documentation (induction, assessments, positioning) • **Cycle 2:** Intubate patient (document airway) • **Cycle 2:** Insert an IV line (document) • Document medication administration • Stop anesthesia	• Consent forms • Review documentation efficiency and content • Avatar functionality for lines and airways • Ease of documenting medications
**Intra-op Nurse** (Cycle 1 *n* = 2) (Cycle 2 *n* = 4)	• Document surgical counts (initial and closing) • Move the patient into the Operating room theater • Complete the timeout checklists • Complete the intra-procedure documentation (site prep, positioning, **Cycle 2:** equipment electrosurgical grounding pad placement) • Document the intra-op medication administration by the surgeon	• Patient movement • Content and build of the various forms (count, site prep, positioning, checklists) • Medication administration workflows
**Post-op Nurse** (Cycle 1 *n* = 2) (Cycle 2 *n* = 4) *participants completed both pre-op and post-op nursing tasks	• Update patient location to the PACU and assign the patient a bed • Release the orders • Remove the IV line • Complete post-op documentation (i.e., vitals, exam, inventory of patient belongings) • Complete discharge education and prepare the patient for discharge	• Patient movement • Content and flow of the assessment forms • Discharge process

The scenarios created by the study team were registered in the test EHR environment by IT analysts proactively. Each scenario was modified to the appropriate steps in overall workflow based on each participant’s role (e.g., booked for a clinic consult visit prior to the ORL sessions). Following each usability test, a debrief was performed at the end of the session using the PEARLS for systems integration debrief framework [30] which included a reactions phase followed by open ended questions to elicit feedback on the system usability including perceived risks and benefits, efficiencies lost or gained, functionality, terminology, patient safety concerns, staff experience, and workflow alignment. All potential hazards and improvement ideas were summarized and cross checked with participants prior to closing each session.

### Simulations

In situ *SFS* sessions were conducted in two sites (main/community hospital) (Fig. 1). Table 2 describes the expanded simulation objectives to include broader system elements such as the implementation of the EHR into the peri-operative environments, processes, and integration with all roles (Table 2).

**Table 2:** Simulation roles, workflow steps, and objectives: Tympanostomy with tonsillectomy & adenoidectomy

**Role (# participants)**	**Workflow steps**	**Objectives for all tools/technology, people, tasks, environment, organization, process ** [24]** sample objectives**
ORL Surgeon (6) Physician Assistant (9) Nurse Practitioner (3) Clerk (1)	• Patient arrives for a surgical consult visit **(Otolaryngology Clinic Location)** • Patient is checked in • Vitals documented (height, weight, allergies) • Place case request for surgery (2 procedures) • Place pre-op and post-op orders for the appropriate phase of care • Obtain surgical consent (Physician Assistant, Surgeon, and patient representative) • Update surgical consent	• Tools and technology to allow for concurrent workflow between Physician Assistant and Surgeon (i.e., orders/consent) • Efficient processes and workflows with the EHR • Communication with patients and families • Environmental impacts related to EHR workflow (documentation in patient room versus clinician office)
Pre-op Nurse (4) -participants completed both pre-op and post-op nursing tasks	• Moves patient into the Pre-op department within the system & assigns them a bed • Release signed and held orders • Complete pre-op documentation (i.e., vitals, head to toe, patient belongings) • Review consent form • Complete the pre-op checklist	• Documentation efficiency • Concurrent documentation with Anesthesia or Surgeons • Roles and responsibilities for patient movement and other tasks
Anesthesiologist (3) Nurse anesthetist (2)	• Review the history • Review the pre-op documentation and pre-evaluation • Place post-op orders for recovery • Create the Anesthesia note • Obtain consent • Move patient into intra-procedure • Anesthesia Checklist • Intra-procedure documentation (induction, assessments, positioning) • Intubate patient (document airway) • Insert an IV line (document) • Document medication administration • Stop anesthesia	• Documentation efficiency • Tools and technology to inform others of Anesthesia tasks (i.e., IV lines and airways) • Ease of documenting medications • Concurrent documentation by Nurse anesthetist and Anesthesiologist • Roles and responsibility change for orders placed by anesthesia • Environment—Portable computer workstations for anesthesia and all roles placement
Intra-op Nurse (6)	• Document surgical counts (initial and closing) for both procedures • Move the patient into the Operating Room • Complete the timeout checklists • Complete the intra-procedure documentation (site preparation, positioning, equipment: electrosurgical grounding) • Document the intra-op medication administration by the surgeon	• Staff shared awareness of patient readiness for surgery • Roles and responsibilities for patient movement and other tasks • Documentation efficiency for quick turnaround cases • Tools for medication administration workflows in the Operating Room
Post-op Nurse (6)	• Update patient location to the PACU and assign the patient a bed • Receive handover from the Anesthetists • Release the signed and held orders • Remove the IV line • Complete post-op documentation (i.e., vitals, head to toe, patient belongings) • Complete discharge education & prepare the patient for discharge	• Roles & responsibilities for patient movement and other tasks • Handover process and tools • Documentation efficiency • Concurrent documentation with Anesthesia or Surgeons • Discharge process

Each session included a systems-focused pre-brief and debrief including added details of in-situ considerations, psychological safety importance [43], and the feedback collection and reporting process. During the debrief, any feedback that was deemed critical to patient safety, regulatory compliance, or operational effectiveness was escalated to the EHR implementation team for urgent review. Consents were obtained for photographs/video capture.

The “surgical consult visit” simulation was conducted in the in situ ORL clinic on a separate day to look at the integration of the EHR in the clinic environment (i.e., arrive the patient, documenting the exam, place the required orders, gather consent). A patient and parent from the institution’s family advisory council participated in this simulation.

The procedure phases of the simulation (i.e., pre/intra/post-operative care) were conducted in two different operating room (OR) locations on two separate days as per the plan for EHR implementation at two locations within the healthcare organization. During the in situ OR days, two standardized patients participated (i.e., patient/parent roles) pre-operatively and post-operatively to enable documentation and consent during patient care. During intra-operative simulations, a Laerdal 5-year-old mannequin was utilized to enable medical interventions, such as medication administration and airway support, including induction, to be completed during use of the EHR.

All findings and recommendations from usability and simulation sessions were summarized and reported to the project team by the consultant group and followed each cycle of testing for review and action by the EHR implementation team. The hierarchy of intervention effectiveness was used as a foundational framework to reflect upon the various recommendations that were established (by participants, facilitators, HF/SFS consultant group) and their effectiveness to address the issues [44].

### Participants

Key leaders from each peri-op stakeholder group identified participants for all sessions based on reasonable criteria determined by the project team. Participants who had prior interface exposure with the target EHR through past work or participation in the organizations EHR implementation working groups were recruited. In cases where a participant was not familiar with the EHR, a project team facilitator guided the participant through an EHR orientation at the beginning of the session.

## Results

A total of 324 recommendations were made during the two usability testing cycles. Table 3 presents the total number of recommendations that were made in each cycle of usability testing and a breakdown of each type of recommendation. The categories of recommendations fell into four potential areas: (1) changes to physical layout, content, and build of the software system; (2) process or workflow changes requiring change management; (3) improvements to the content for training materials and education sessions; and (4) other items which could include physical hardware and further investigations.

**Table 3:** Usability testing cycle 1 and 2: number of recommendations categorized with select examples

Recommendation Type	# of Recs: Cycle #1	# of Recs: Cycle #2	Select examples
Change management/process change	15	10	• **Weight-based medication ordering and administration:** site standard to avoid placing weight-based medication orders in advance of the surgery to avoid patient safety risk of weight changes resulting in dosage errors. Process of capturing patient weight in the system prior to ordering and administration of weight-based medications • **Change in process** to document information and place orders during the visit instead of dictating after the visit was completed • **Roles and responsibilities** for ordering and documentation (e.g., which role charts the lines or tubes intra-op may change from current process) • **Coordination of workflows** using system between the different scheduler and clinical roles • Standardizing what is required versus auxiliary documentation fields
Training and education	10	19	• **New terminology**: Definition of new terminology users didn’t understand (e.g., “patient class categories”) • **Clarify and educate** on the how to schedule complex procedures (i.e., multiple procedure surgical cases) or reschedule surgical cases • **Charting by exception**: there are too many unnecessary fields in the forms and flowsheets to accommodate other care areas/patient populations, determine what is the required documentation for these cases • Training for Physicians on how to personalize their order sets and tools to improve efficiency
Software/build change	91	175	• **Consents**: usability issues with design, and flow of the consent forms (e.g., some of the patient representative roles listed cannot give consent; information entered in the consent fields were not populating onto the consent form) • **Aligning content/terminology** to current workflows & practices (e.g., otorrhea instead of drainage) • **Re-organizing tools** intuitively to match workflow (e.g., flow of the forms to match the sequence they will be filled out in the workflow; commonly used items or fields should be first) • **Re-design of order sets: Otolaryngology** pre-procedure order sets were confusing, orders for intra-procedure administration were within the pre-procedure categories; the default dosages and frequency did not match the established workflows; terminology not common to users (e.g., non ORL terminology), titles not matching content of order • **Pre-procedure checklist:** changes to reflect roles and responsibilities for specific items prior to surgery • **Building efficiency and reducing click fatigue** (e.g., developing ordering tools with required defaults pre-selected or quick documentation tools to allow one click for all “normal parameters” to be selected versus five clicks to individually select them all; Patient positioning templates need to be accurately built for quick turnaround cases) • **Intra-procedure checklist/timeouts** were inefficient (e.g., 3 separate checklists that required intra-op nurses to enter a username and password as part of the documentation to finalize each checklist). The initial build included duplicative information across the checklists; other roles were responsible for some of the items that they were attesting to, so should be removed from the intra-op nurse checklist • Building coordination/awareness between various team members • **Medication naming and dosing** defaults should match site standards (e.g., ibuprofen set to 10 mg/kg, every 8 h PRN but site standard is 5 mg/kg, every 6 h PRN) • **Alerts:** (e.g., changes needed to avoid alert fatigue, capture critical errors such as weight change, max dosage alerts) • **Date fields:** too many undefined date fields for booking a surgery, it was not clear to users as to what each field was referring • **Search terms and synonyms:** Adding commonly used search terms as synonyms to improve searching tasks (e.g., “ENT” and “ORL” for otolaryngology service) • **Removing mandatory fields:** or “hard stops” that were confusing to users or not necessary to be mandatory (e.g., documenting the count was correct for the initial instrument count) • **Build Misconfigurations: Medication/prescription:** (e.g., adjusting a medication duration time or dosage for a discharge prescription didn’t automatically update to a new quantity to fill) • **Patient Belongings:** selection options in these forms should align with age appropriate for pediatric site, not adult focused items (e.g., dentures may not be the most frequently documented item, so should not be located at the top right of the list. Toys or sippy cups are often brought with the patients so they should be added to the options available
Other	4	0	• Purchasing IT equipment (signature pads required to complete consents) • Investigating downstream effects with different tools for Anesthesia providers to document with compared to other clinicians (e.g., to document airways in a note rather than the tool used by other clinicians, can the other clinicians see that information in their screens?)
**Total # of recommendations per cycle**	**120**	**204**	

**Fig. 2:**
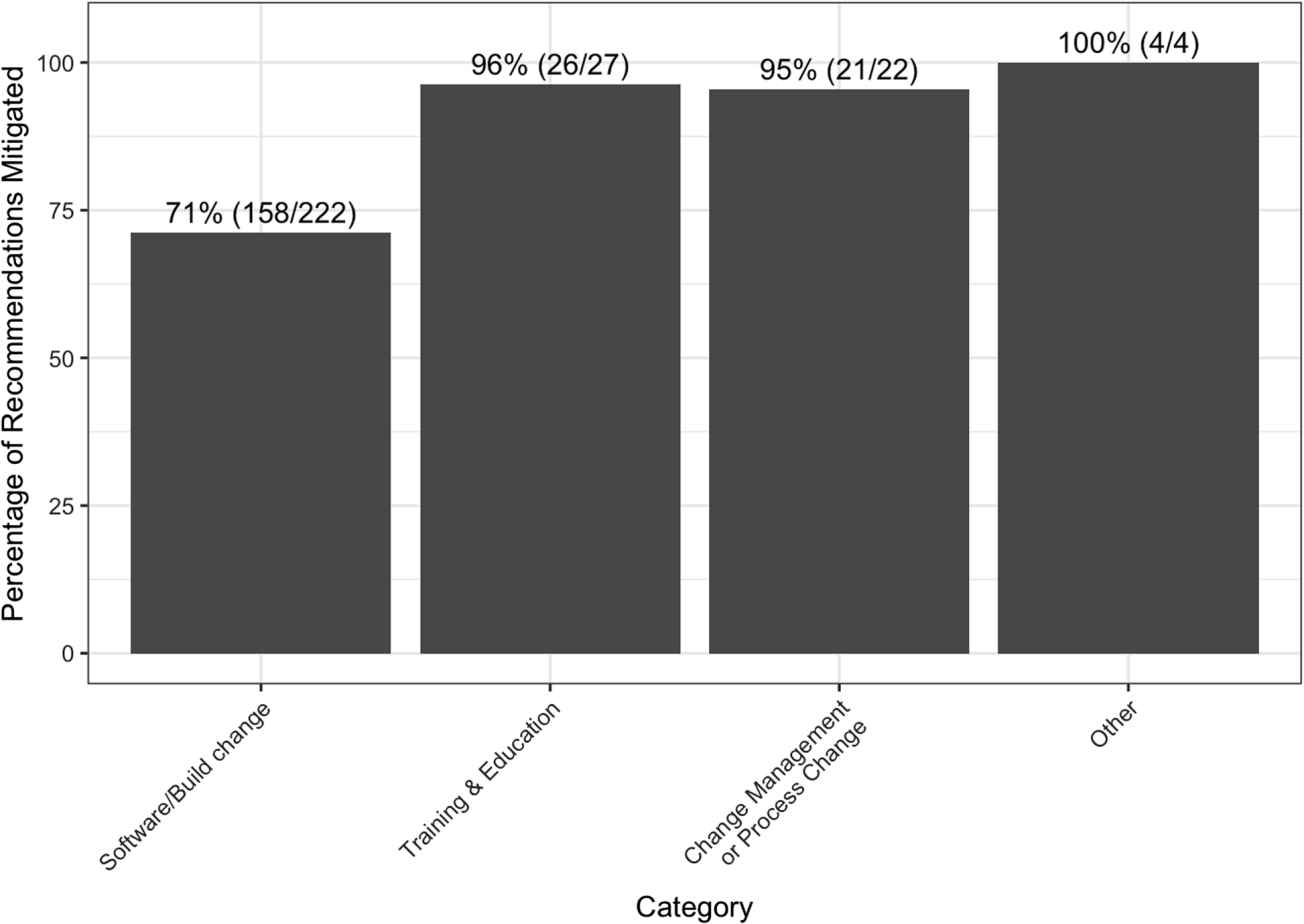
Total percentage (and count) of recommendations mitigated from usability 1 and 2 combined

Of the 324 total recommendations that resulted from usability testing, there were 275 unique recommendations between usability cycle 1 and 2 of testing. Of those, 76% (209) were completed within six months post go-live. Nearly all outstanding recommendations involved software or build changes. Half of these stemmed from technical limitations or organizational decisions not to adopt the recommendation that resulted from the usability testing. For example, the organization standardized advanced scheduling timeframes for surgery, which differed from previous categorizations, and certain surgery-specific questions could not be moved to different forms in the workflow due to technical system constraints. Other unaddressed recommendations were deferred based on time and resource constraints (Fig. 2).

### Recommendation category

Tables 3 and 4 describe examples of specific findings from both the usability testing and simulations as they relate to optimal outcomes. Efforts were made to prioritize resolution strategies based on systems focused solutions, when possible, versus only people focused (e.g., training) solutions. Sample critical outcomes include improving efficiency for documentation and ordering to minimize surgical start delays and making the system more intuitive by matching staff workflows to improve safety and satisfaction (e.g., improving the design of the electronic surgical consent forms).

**Table 4:** SIEPS 2.0 System simulation recommendations with select examples

SEIPS system elements and brief descriptions	# Recommendations	Select examples
Tools/Technology Consider usability, functionality, level of automation etc.)	117	• **Electronic Consents**: Update consent form build to improve usability & align with workflows. *Risk uncovered for potential wrong site surgery through ongoing consent build issues* • **Visibility of completed consents:** intra-op nursing requires easier access to view consents during timeout (e.g., add to main side bar for their view) • **Ready for Procedure Status:** indicators only showing for RN roles, other roles such as Anesthesia would benefit from this information. The surgeon ready for procedure also needs to update automatically or allow for manual update by the surgeon or physician assistant • **Alert Notices**: Ensure alerts are firing properly (e.g., weight-based medication alert should not fire for non-weight-based medications) • **Fire risk score:** incorrect calculation of fire risk rating based on the current build, it is not aligning with hospital policy (e.g., calculating a lower fire risk score than they currently indicate) • **Chart locking finding**: patient chart locked with concurrent documentation from different roles. Issue with aligning to current workflows that require access, especially in these quick turnaround cases • **Pain Scales**: incorrect pains scales were included in the build and do not match the established one • **Aligning Language**: e.g., change to surgical incision versus surgical “wound”; different roles show “case start” versus “procedure start” times • **Positioning Templates**: not correct for the default positioning that would be used in these cases • **Missing key fields**: e.g., FiO2 and EtCO2 data streaming for Anesthesia views; patient weight missing on the Anesthesia intra-procedure screen, e.g., order sets missing key medications • **Weight-based medication rounding:** system not rounding to appropriate amounts for administration (e.g., 12.49 mL of oxycodone or 311.3 mL of acetaminophen)
Organization Consider staffing, workload, schedules, education and training, work culture etc.)	6	• Systematic review of all previous usability recommendations (i.e., internal process to review and address usability findings prior to simulations) • **Staff Comfort/Education**: participants requested additional simulations sessions to practice workflows prior to launch • **Staff requests**: Increase staffing or reduce surgical case load in the initial launch period while learning • **Super-Users:** request they are only educators during launch and not assigned patient care duties. Super users not feeling prepared due to receiving the same education as all staff (just earlier) • **Patient/Family Portal**: improve communication with parents/families on changes to the patient portal; address upcoming downtime
Environment Consider how environment impacts their role- distractions, layout, space etc.)	5	• **Computer Supply**: Ensuring enough mobile computer workstations for various roles in the Operating Room to chart at the same time (e.g., Anesthesia and Nurse Anesthetist)
Roles/responsibilities/tasks (who and what is required—difficulty, complexity etc.—what do they need to function effectively?)	14	• **New role allocation**: Additions to training for new tasks that users will have to complete (e.g., nursing placing orders in the Operating Room) • **New tasks**: Determine who is responsible for completing specific tasks in the sequence and ensure all users are aware of the correct workflows (e.g., patient movement/event steps, medication orders), as this is different from current practices
Process What processes are impacted?- Select examples	9	• **Uploading Paper Consents**: Clarify workflows for paper consents to be uploaded into the EHR. Note: consents can be attached to case even with missing signatures, posing a risk • **Clinical Event Debriefings**: Staff ask to initiate clinical debriefings during launch to aid in sharing learnings • Ensure that placing discharge orders in advance does not impact the subsequent care areas ability to complete their tasks (e.g., PACU nurses must be able to action post op orders and complete their documentation tasks) • **New process between surgeon and physician assistant in Otolaryngology clinic**: Develop a workflow for ordering and consent signing in high throughput clinic using the HER
Total # of recommendations	**151**	

Systems focused simulations revealed 151 recommendations, the majority of which related to additional build changes. Critical recommendations such as the electronic consents were escalated to the EHR implementation team for urgent review.

## Discussion

The ability to prioritize patient safety with proactive testing of new workflows requires a significant shift in an organization’s thinking and process. It requires allocating limited resources and time to avoid the commonplace, often a more reactive, higher risk post-implementation of changes and mitigation for poorly designed systems.

Human factors and SFS methods are proactive approaches to identify risk, mitigate harm, and improve efficiency while embracing user-informed design [43]. Through two cycles of usability testing followed by in situ simulations, we studied the design and integration of all system elements prior to the EHR launch which resulted in over 400 unique recommendations. This approach allowed us to identify high risk and high impact findings early in the project, allowing for more time to mitigate identified hazards and better align the design and adoption with users, prior to implementation.

The findings and outcomes found include a range of usability and system issues including latent safety threats and their impact on safe and quality patient care. In our project, there were a plethora of usability improvements, including some critical issues that were uncovered and mitigated prior to the go live date. Examples included considerable user concerns with consent forms and processes (e.g., risk of wrong site surgery due to consent design issues, incomplete consents), system misconfigurations caught early that would have resulted in incorrect medication quantities being ordered, and poor alignment with users’ workflows that resulted in observed delays and user frustration. Improving the patient experience included adding a field to electronically track patient belongings in the OR. Considering the typical length of an ear tube surgery (i.e., < 10 min per case), with a rapid turnaround and high volume per day, the importance of reducing unnecessary clicks, ensuring that positioning templates for surgery are aligned to the typical patient case, and EHR forms that are aligned to workflow cannot be overstated. Time efficiencies to be gained, user satisfaction (or frustration), and unnecessary time pressures in an operating room environment are all outputs of EHR design and how well it is optimized to the context. Additional benefits were reported, prompted by the pre-implementation usability tests and simulations, where additional team-based simulations were set up by the end users to further practice using the EHR and workflows while moving away from previous siloed training approaches.

Our methods are applicable to other institutions who are interested in adopting more proactive patient safety approaches such as HF usability testing followed by SFS in Plan, Do, Study, Act cycles of testing with new EHRs. The active engagement of users early from needs assessment through to project completion enables system learning of how work is actually done within the new software [45]. This is often a missing link for organizations to realize higher reliability, safer care, and a health system that is well designed and supports people to do their best work [46]. Bates et al. [47] describes decision support when combined with an EHR a potent means to create a “better cockpit” for clinician behaviour and patient outcomes to help them avoid errors, be more thorough and align better with evidence based practice. They emphasize the inherent need for HF usability testing to make it easier for the clinician to “do the right thing” and that the system’s design can make the difference between success and failure to adopt. This evidence builds on our use case to ensure these methods are utilized prior to launch of new EHRs as demonstrated in a peri-operative environment.

The hierarchy of intervention effectiveness [44] is a risk management theory that defines interventions to reduce risk (i.e., recommendations for change) into people focused and systems focused interventions [48]. Systems-focused changes such as forcing functions, introducing automation, and standardization or simplification of processes and tools are more effective at changing human behavior compared to people-focused interventions that include policy development and training and education. Our project resulted in a wide variety of recommendations spanning both the system and people focused elements. As we were early in the EHR build, the majority of recommendations made were for changes to simplify and standardize the software/build to improve automation, more effective at improving compliance specifically from a safety science lens, compared to the education or policy type strategies [47, 49]. Testing the technology through 1:1 usability testing first was helpful to focus on the elements of the specific tools within the EHR and make specific build changes that otherwise may have been challenging to identify if only simulation was used. Combining our approach of the usability testing followed by simulations enabled deeper understanding and testing of the system elements systematically resulting in a broad range of recommendations to improve the integration of the EHR into the health system.

### Lessons learned/reflections

Our project was intended to utilize proactive testing prior to the EHR launch to identify issues and safety threats before using in the live clinical environment for the first time given the high risk and large amount of change happening at once. Shifting safety upstream is essential if our goal is to become more proactive versus reactive to harm in healthcare. Using routine testing at this early juncture was helpful to uncover many of the listed problems that enabled our ability to fix them prior to use in the live patient care environment. Further, application of HF and SFS during EHR design changes, upgrades, or even an uptick in EHR reported safety threats, should be a part of routine operations to test any new or potentially concerning changes to avoid inadvertent harmful, ineffective, or insufficient modifications to an existing EHR.

Our project had limitations, some of which were not predictable. Our timelines were short and with the large volume of findings uncovered (i.e., 475 recommendations), this required organizational resources to mitigate risks in a timely way before the next cycle of testing could proceed. While all recommendations were reviewed, the decision as to whether to implement a recommended change was left to the multidisciplinary EHR implementation teams. As such, it was not always clear what changes the various analyst teams had made in the IT testing environment prior to the next cycle of testing. Although limiting, this was not reported to have a negative impact on the effectiveness of subsequent cycles of testing; users and faculty were pleased to proceed as long as the facilitators and users could see progress being made toward improvements. A purposeful anticipation for the unknown risks that may surface, and a process for escalating the critical ones was key to enable teams to make iterative improvements in real time during simulations (e.g., consent form issues were escalated immediately by leadership for mitigation during the simulations). Immediate escalation by people with the ability to make changes, especially when timelines are tight cannot be understated. This occurred multiple times during our project when high risk findings required escalation by leadership.

Ensuring that sufficient time is allotted in project planning is essential to effectively execute each cycle of iterative testing. Unfortunately, no perfect timeline can be provided as a “catch all” given every project is variable in its scope, overall project timeline and budget, number of evaluation objectives that are identified as well as number of cycles of testing that are required. However, building usability testing and simulation into the project timeline and ensuring availability of resources such as IT analysts to build patients for the testing, and to address recommendations and outcomes within their workload is beneficial to reducing the impact on project timelines. Dubé et al. [23] describe the essential need for a well thought out and executed “pre-work” phase to building impactful system focused simulations as a reference for project planning considerations [50].

Given the limited resources, not all areas of the EHR could be evaluated with HF and SFS. Without advertising, multiple users came forward from other clinical areas of the hospital requesting similar testing of workflows in their area during design. This is indeed a gap in the traditional IT project implementation planning. Resources for conducting these methods can be limited when implementing a new institution-wide EHR. The most challenging aspect of this work was the inability to offer these methods to all interested groups who requested it, once the work was underway. The need to carefully prioritize is important and difficult. To prioritize, we determined that areas where high risk/time pressured documentation during live patient care and where efficiency and high workload velocity were critical, would most benefit from this evaluation. In this project, the high volume of cases and high-risk environment of the operating room and the fact that lessons learned could be applied in other operative settings (i.e., beyond the ear tube cases to other surgical situations), made the perioperative setting a priority for HF and SFS testing. We knew based on time limitations and resource constraints that we would need to limit the patient care areas included and chose to prioritize those areas and workflows where there was a high level of patient care interface with the EHR, and where patient harm could be most at risk. A future direction would be to further study which aspects of a new EHR would benefit the greatest.

This demand for HF and SFS is a marker for the increasing need to embed HF and systems simulation specialists into healthcare organizations. As we strive for a safer and more reliable healthcare system, we advocate for organizations to put their resources towards more proactive patient safety testing whenever possible.

Our paper demonstrates a QI use case highlighting the essential need for a proactive and synergistic use of HF and SFS methods during the implementation of eHealth technologies with peri-operative end user teams. Our project resulted in a total of 475 recommendations to improve the design and adoption of a new EHR system at a US pediatric hospital.

## Data Availability

Data is provided within the manuscript.

## Abbreviations

IT: Information technology
EHR: Electronic health record
HF: Human factors
SFS: Systems focused simulation
SEIPS 2.0.: Systems Engineering Initiative for patient safety
QI: Quality improvement
ORL: Otolaryngology
